# Metasurface on integrated photonic platform: from mode converters to machine learning

**DOI:** 10.1515/nanoph-2022-0294

**Published:** 2022-07-20

**Authors:** Zi Wang, Yahui Xiao, Kun Liao, Tiantian Li, Hao Song, Haoshuo Chen, S. M. Zia Uddin, Dun Mao, Feifan Wang, Zhiping Zhou, Bo Yuan, Wei Jiang, Nicolas K. Fontaine, Amit Agrawal, Alan E. Willner, Xiaoyong Hu, Tingyi Gu

**Affiliations:** Department of Electrical and Computer Engineering, University of Delaware, Newark, DE 19711, USA; Physical Measurement Laboratory, National Institute of Standards and Technology, Gaithersburg, MD 20899, USA; Peking University, Beijing 100871, China; School of Electronic Engineering, Xi’an University of Posts & Telecommunications, Xi’an 710121, China; Department of Electrical & Computer Engineering, University of South California, Los Angeles, CA 90089, USA; Nokia Bell Labs, 600 Mountain Ave, Murray Hill, NJ 07974, USA; Department of Electrical and Computer Engineering, Rutgers, The State University of New Jersey, Piscataway, NJ 08854, USA; College of Engineering and Applied Sciences, Nanjing University, Nanjing 210093, China; Department of Physics & Astronomy, University of Southern California, Los Angeles, CA 90089, USA

**Keywords:** deep learning, metasurface, silicon photonics

## Abstract

Integrated photonic circuits are created as a stable and small form factor analogue of fiber-based optical systems, from wavelength-division multiplication transceivers to more recent mode-division multiplexing components. Silicon nanowire waveguides guide the light in a way that single and few mode fibers define the direction of signal flow. Beyond communication tasks, on-chip cascaded interferometers and photonic meshes are also sought for optical computing and advanced signal processing technology. Here we review an alternative way of defining the light flow in the integrated photonic platform, using arrays of subwavelength meta-atoms or metalines for guiding the diffraction and interference of light. The integrated metasurface system mimics free-space optics, where on-chip analogues of basic optical components are developed with foundry compatible geometry, such as low-loss lens, spatial-light modulator, and other wavefront shapers. We discuss the role of metasurface in integrated photonic signal processing systems, introduce the design principles of such metasurface systems for low loss compact mode conversion, mathematical operation, diffractive optical systems for hyperspectral imaging, and tuning schemes of metasurface systems. Then we perceive reconfigurability schemes for metasurface framework, toward optical neural networks and analog photonic accelerators.

## 1 Introduction

In recent years, photonic integrated circuits (PICs) have emerged as one of the key platforms for components in optical communications and computing. Especially, the expanding of components libraries in silicon photonics with verified performance through manufacturing manifests the complexity, functionality and scalability of PIC system [1]. Most of those components available in foundry process design kits are designed for fundamental mode only, because of the unacceptable crosstalk between higher order modes and loss due to the multimode interference. However, the high refractive index difference between the silicon core and silica cladding layer makes a very strong mode dispersion in silicon photonics, which make it possible to utilize multimode in PICs [2]. Parallel to the efforts on developing multi-mode waveguides-based components by profile trimming or inverse design, here we review the utilization of metasurface design concept for diffraction-based PICs. The diffractive PIC breaks the waveguides confinement designed for single or few modes waveguide, but builds on the ‘free-space propagation’ in silicon-on-insulator (SOI) slab waveguide.

Metasurfaces are planar photonic elements, composed of subwavelength distributed antennas with spatially varying geometric parameters, able to control the propagation of light at will [3]. Metasurfaces can be used for different applications, for example, focusing and holographic imaging. Meta-system can also be realized by cascading multiple layers for metasurfaces, which can be used for more complex applications, such as analog computing and spectroscopy. Most of the metasurface works have focused on controlling the light propagation in free-space, however, besides free-space metasurfaces, they can also be used for integrated photonics, to control the guided multimode in waveguides [3, 4]. Multimode waveguides increase the data throughput, and the diffraction and interference that occurs in multimode waveguides make it possible for optical signal processing. Compared with traditional methods, metasurface-based design method is focused on utilizing the diffraction of light, which is especially suitable for applications such as Fourier transform, analog signal processing, and convolution. All these applications require sufficient space for ‘free-space propagation’ in slab waveguide, and thus cannot be achieved using compact inverse design approaches.

In this paper, a review of recent progress in metasurfaces on integrated photonics platform is given. The design principle of high contrast transmitarrays (HCTAs)-based metasurfaces is discussed in Section 2. Mode converters based on metasurfaces are discussed in Section 3. In Sections 4 and 5, we discuss a meta-system that is used for analog computing and machine learning. In Section 6, we discuss the perspectives for integrated metasurfaces.

## 2 Design principle of integrated meta-atom/metasurface cell

**Figure 1:**
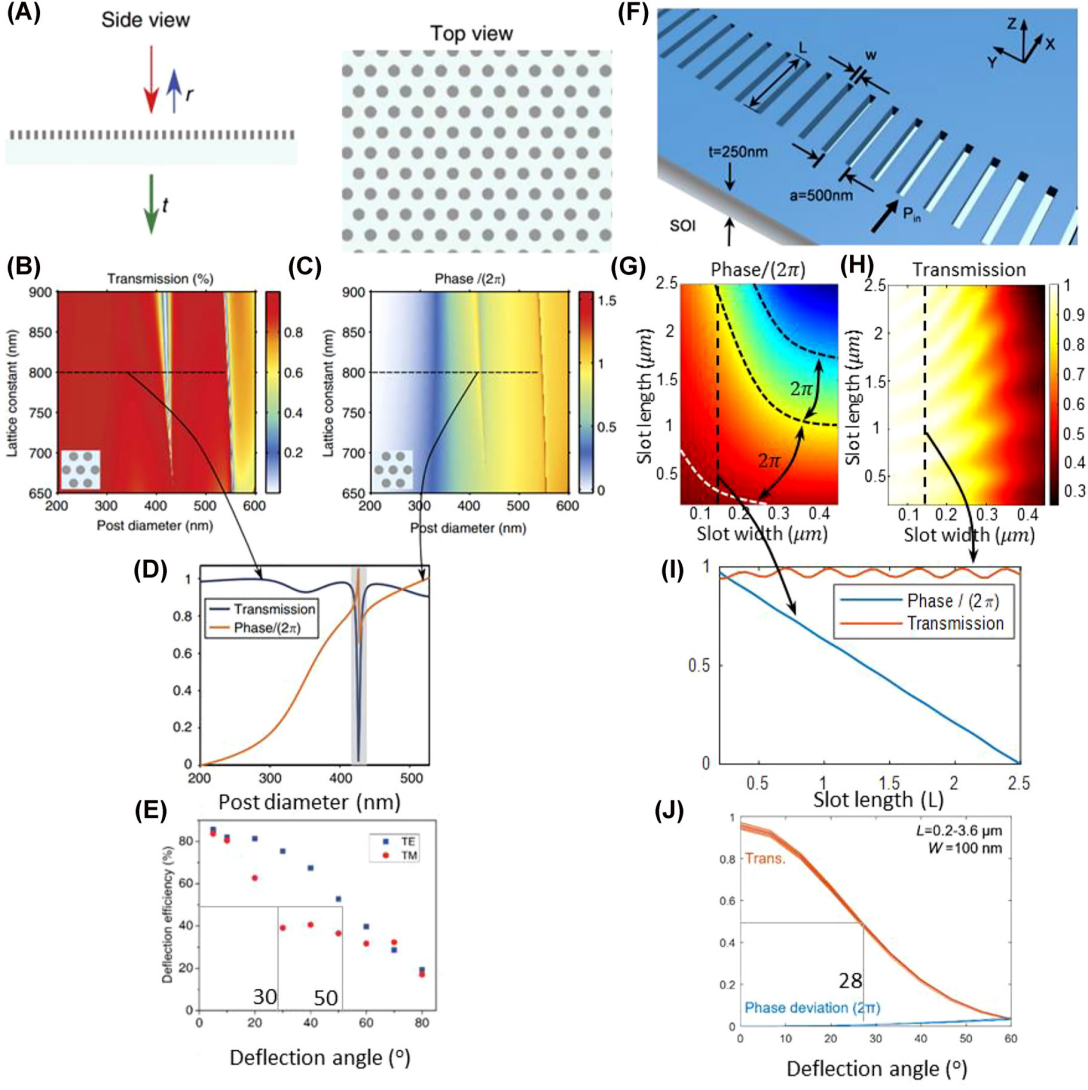
Free-space HCTA inspired on-chip metasurface phase array [5, 6]. (A) The schematic representations for free-space HCTA metasurface. (B) Transmission and (C) phase shift versus post diameter and lattice constant [5]. A zero to 2*π* phase shift and transmission above 90% can be achieved simultaneously. (D) Simulated transmission and phase of the transmission coefficient for a family of periodic hexagonal HCTAs with lattice constant of 800 nm, and varying post diameters. (E) Transmission and phase deviation versus incident angle and slot lengths of the free space 2D HCTA metasurface. (F) Schematics of the integrated metasurface defined in silicon-on-insulator substrate (SOI). (G) Phase shift and (H) transmission versus slot width and slot length. A zero to 2*π* phase shift and transmission above 90% can be achieved simultaneously for on-chip HCTA metasurfaces. (I) Transmission and phase versus slot lengths with slot width set as 140 nm. (E) The deflection efficiency of the free-space periodic HCTA. (J) Transmission and phase deviation versus incident angle and slot lengths of the on-chip HCTA metasurface. The figures are reproduced with permission. (A)–(E) Ref. [5] © 2015, Nature Publishing Group, a division of Macmillan Publishers Limited. All Rights Reserved. (F)–(J) Ref. [6] © 2019, The Author(s).

The design of metasurface for integrated photonics platform follows the same rules as free space metasurface. By changing the geometric parameters of subwavelength unit cells, the amplitude or phase of the transmitted or reflected light is changed with subwavelength resolution. Arbabi et al. [5] designed a free space two-dimensional metalens with high numerical aperture based on HCTA, as shown in Figure 1(A). Figure 1(B) and (C) show the simulated transmission and phase shift of the HCTA structure versus post diameter and lattice constant. Figure 1(D) shows the transmission and phase shift of periodic hexagonal HCTAs with lattice constant of 800 nm, and varying post diameters. Figure 1(E) shows the deflection efficiency versus different deflection angle of the HCTA. Inspired by this free space HCTA metasurface, Wang et al. [6] designed an on-chip one-dimensional (1D) HCTA for analog computing. The HCTA is composed of 1D air slot array defined on the silicon slab waveguide, as shown in Figure 1(F). The phase shift and the transmission of the guided wave can be modified by changing the geometric parameters, the length and width, of the air slots, as shown in Figure 1(G) and (H). Figure 1(I) shows the transmission and the phase shift of the 1D HCTA versus slot length, with a fixed slot width of 140 nm. The large refractive index difference between silicon and air ensures light confinement within the slots. The effective refractive index of the guided wave in the slot and slab waveguide is different, which allows phase shift of the transmitted light to be controlled by varying the slot length. For the same incident angle, the deviation of the transmission and phase is small for different slot lengths, as shown in Figure 1(J). A typical diffractive surface is made of nanostructures with a fixed depth (*z*-direction) as the direction of the input light, and the topologies and dimensions in-plane (*x–y* directions) are designed to form one-to-one correspondence with the complex transmission coefficient through numerical simulations. Alternatively, the wavefront of in-plane guided wave can be controlled by one-dimensional metasurface defined in the thin film. The integrated metasurface allows the variation of the nanostructure length along the direction of light propagation. On widely used silicon-on-insulator substrates, the high refractive index contrast allows 2*π* phase shift of the input wave within ≈1 µm trench length variations. With such design principle, one can design the integrated metasurfaces to achieve almost arbitrary complex transmission for different applications.

## 3 Ultra-compact, low loss and broadband mode converters by integrated metasurface

Photonic integrated circuits offer an attractive platform but still facing significant challenges, including miniaturizing device footprints, increasing device operation bandwidth and robustness, and reducing device insertion losses [7–10]. Integrated optical waveguides with gradient metasurface structures can help address some of these challenges. Meta-structures have emerged to control guided waves [11–15] and couple guided waves with waves propagating in free space [16–18]. They are arrays of sub-wavelength structures that can apply locally and spatially varying phase shifts to transmitted or reflected electromagnetic (EM) waves [19–21]. The gradient change of the nanostructure (for example, antenna shape, size, and orientation) in the sub-wavelength thin layer can manipulate the out-of-plane EM wave in free space, resulting in the shift from the simple components of the micro flat lens [5, 22–24] and the hologram [25–27] to the more complex analog signal processing system [28, 29] and spectrometers [30]. Especially, metasurfaces can convert on-chip guided mode to single and multiple beams with desired polarization, directionality and divergence, which provides exceptional robustness toward meeting the goal of on-chip atom trapping for quantum sensing applications [31].

**Figure 2:**
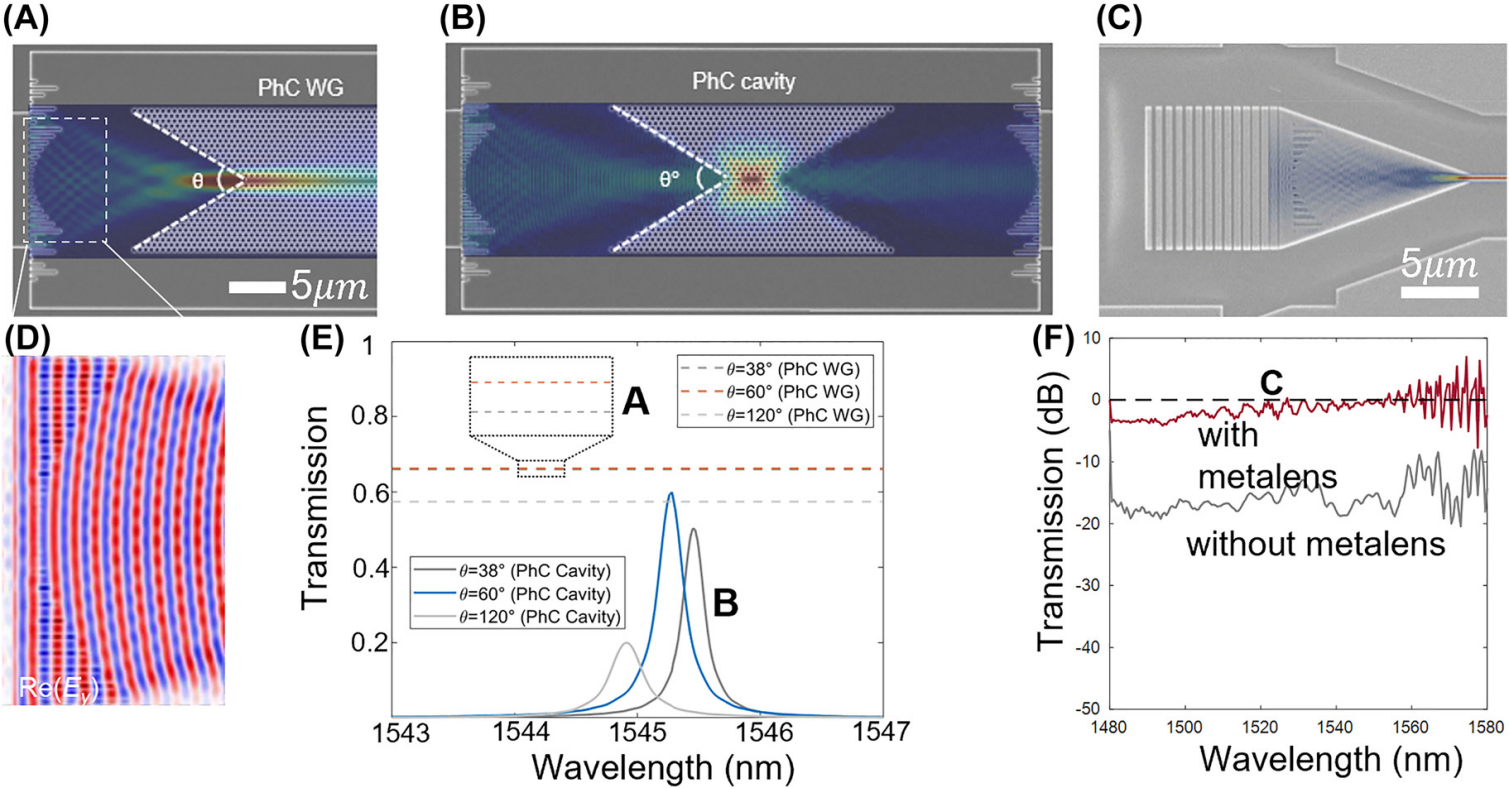
Broadband and low loss mode size conversion with gradient transverse metasurface. (A) Device geometry of metalens – PhC WG system with light distribution of |Ey|^2^ on the focal plane. (B) Device geometry of metalens – PhC L3 cavity – metalens system with mode profile at resonance. (C) The SEM image of the metalens enabled ultracompact low loss taper. (D) Zoom-in view of the simulated electrical field distribution of the HCTA based metalens in A–C. (E) Average transmission spectra of the metalens – PhC WG system in (A) and peak transmission of metalens – PhC single modecavity – metalens system in (B), with the PhC coupler angle of 38°, 60°, and 120° [33]. (F) Measured transmission spectra of the device in C (red), compared to the same taper design without metalens (gray) [6]. The figures are reproduced with permission. (A), (B) and (E) ref. [33] © 2021, The Author(s). (C), (D) and (F) ref. [6] © 2019, The Author(s).

**Figure 3:**
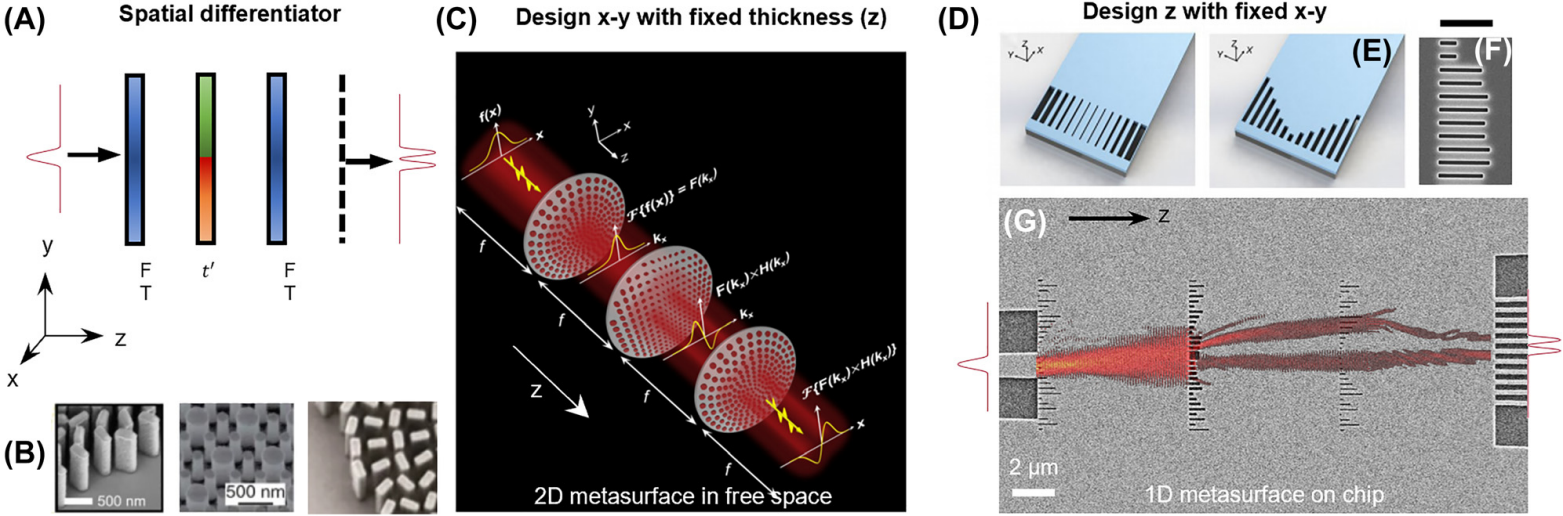
Fourier optic implementation of a spatial differentiator. (A) Schematics of a three-layer spatial differentiation system for diffraction. (B) Scanning microscope images (SEMs) of dielectric [f1] and metallic metasurface; the transmitted wavefront can be designed towards multi-layer metasurface systems. (C) Schematic illustration of a three-layer metasurface system, composed of two metalens for Fourier transformation and middle layer of designed mask [49]. (D) Design of integrated metalens by varying lattice constant and (E) trench length [50]. (F) SEM image of the 1D metasurface defined on SOI substrate. Scale bar: 1 µm. (G) SEM image of a spatial differentiator based on integrated metasurfaces [6]. © 2020, The Author(s).

The HCTA described in Section 2 provides broadband and low loss mode size conversion within compact dimensions. Xiao et al. designed a low-loss on-chip microsystem based on broadband metalens and a photonic crystal (PhC) resonator [32, 33]. By adjusting the on-chip metalens’ focusing length and mode dimension, the insertion loss between the metalens, the PhC resonator and waveguide structures is minimized through mode-matching. Three different PhC couplers with angles of 38°, 60°, and 120° are designed by removing individual air holes at the two-side edges of PhC to generate an input and output PhC taper [34, 35] (Figure 3A and B). The metalens can be placed on both input and output ports to assist with mode conversion. Figure 2A illustrates the metalens PhC waveguide coupling, and the metalens design allows direct coupling into the PhC cavity. The bilateral metalens system in Figure 3B shows slightly higher insertion loss than the broadband metalens-PhC waveguide design, concerning the tunneling process through the PhC cavity. The mode-size conversion significantly reduces the tapers length connecting the grating coupler and single mode waveguide (Figure 2C). With the integrated metasurface controlled wavefront (Figure 2D), low insertion loss < 1 dB can be achieved within the devices in Figure 2A and B (Figure 2E). The mechanical robustness is desired for improving sensitivity in nanophotonic sensors [36–38] and reducing operation power for PhC based active silicon photonic components. Without the metalens enabled low loss wavefront conversion, an insertion loss of the ≈13.7 µm taper between grating waveguide and single mode waveguide results in ≈18 dB loss (gray curve in Figure 2F). The total insertion loss can be reduced to less than 1 dB from 1480 to 1580 nm. Typical linear table requires more than 200 μm length to achieve similar level of insertion loss as the one in Figure 2C. Other than the transverse metalens described above, gradient ‘longitudinal metasurface’ placed parallel to the wave-propagation direction have been investigated by a number of groups, for inter-mode conversion in a few mode integrated photonic waveguides. The examples are shown in refs. [3, 4].

## 4 Integrated metasystem for mathematical operations

Most of the optical signal-processing systems are based on the Fourier transform property of a lens. Integrated metasurfaces enable arbitrary phase control of the transmitted light, and one can design integrated metalens and build the integrated 4-*f* system to perform analog optical signal processing. Each subwavelength unit-cell of a metasurface can be independently designed, toward the goal of achieving the desired transmission [22, 39] and reflection coefficients [40, 41]. Its amplitude of transmission coefficient can be independently controlled by the trench width or lattice constants. With full control of complex transmission coefficient ‘pixel-by-pixel’, metasurface systems can be designed towards complex spatial information transformations. Beyond one-step etching defined metasurface systems, gray-scale nanofabrication creates periodic and quasiperiodic sinusoidal Fourier surfaces with well controlled wavefront shaping capabilities [42].

### 4.1 Fourier optics and mathematical operation

The Fourier transform capabilities of a lens provide numerous applications in optical signal processing. The patterned surfaces with subwavelength features can interact with incoming beam and the interference of the diffractive wavefronts can serve as conventional lens, where each unit is designed in the way that the amplitude of the transmission coefficient is fixed at ‘1’ and phase needs to be tuned with the nanostructure geometry (also named as ‘phase-only metasurface’). Since the phase and amplitude of the transmission coefficient is usually proportional to the nanostructure dimensions, geometrically gradient variation creates gradient metasurface (e.g., metalens), and abrupt change between adjacent subwavelength unit-cell creates non-gradient metasurface (e.g., spatial Fourier domain filters or advanced functions of machine learning [43, 44]). The prior one performs Fourier transform at focal plane as conventional lens, and the latter one can be inserted at the focal plane as filters in spatial Fourier domain.

One important application of Fourier optics is edge detection [45]. Following the first demonstration of mathematical operation with metasurface [28], spatial differentiation has been implemented in a dielectric nanophotonic slab and metallic plasmonic surface [41, 46–48]. Recent research proposes that the spatial differentiation operator can be implemented with all-metasurface systems, where the first and the third layers of metalens serve as Fourier transformers, and the second metasurface are programed for a desired function *t*′ (Figure 3A). In free space, such metasurface systems can be composed of two-dimensional array of nanostructures with the same height (in *z*-direction) (Figure 3B). Cascaded metalens–metasurface–metalens systems perform spatial edge detection (Figure 3C) [49]. The light propagation in planar waveguides can be controlled by in-plane one-dimensional metasurface. On SOI platforms, the amplitude and phase of transmission coefficient for each pixel is determined by lattice constant (Figure 3D) or trench length (Figure 3E) [50]. Figure 2F shows a top view of non-gradient metasurface for in-plane wave. Without active alignment, the lithography defined system perform one-dimensional spatial differentiation (Figure 3G) [6].

### 4.2 Broadband metasurface convolver

Based on convolution theorem, one can easily achieve convolution using a 4-*f* system. There are various methods for designing on-chip dielectric metasurfaces, including using the effective medium theory to simplify the metasurface structures, transforming the problem of the structural size design into a problem of refractive index distribution in geometric optics [6, 51]; using the transmission matrix to describe the propagation of optical modes, obtaining metasurface structures by optimizing the transmission matrix, or searching the required structure parameters through the target transmission matrix [52, 53]; In addition to above forward direct design methods to obtain required metasurface structures, structural parameters of the metasurface can also be searched by inverse design [14, 15]. Different from the traditional intuition-based parameters scanning, the design scheme optimizes the optimal structure parameters through intelligent algorithms with greater flexibility and controllability to approach the limits of device performance [54, 55]. Convolution [56] is one of the most basic and important operations in signal processing, especially in the field of signal analysis and image processing. Since all-optical convolution operation gives full play to the ability of optical parallel computing [57, 58], it is one of the most promising directions in all-optical computing. Recent research reports a strategy to utilize genetic algorithm (GA) to assist the design of a dispersionless metalens and a nanophotonic convolver based on silicon metasurface [59].

**Figure 4:**
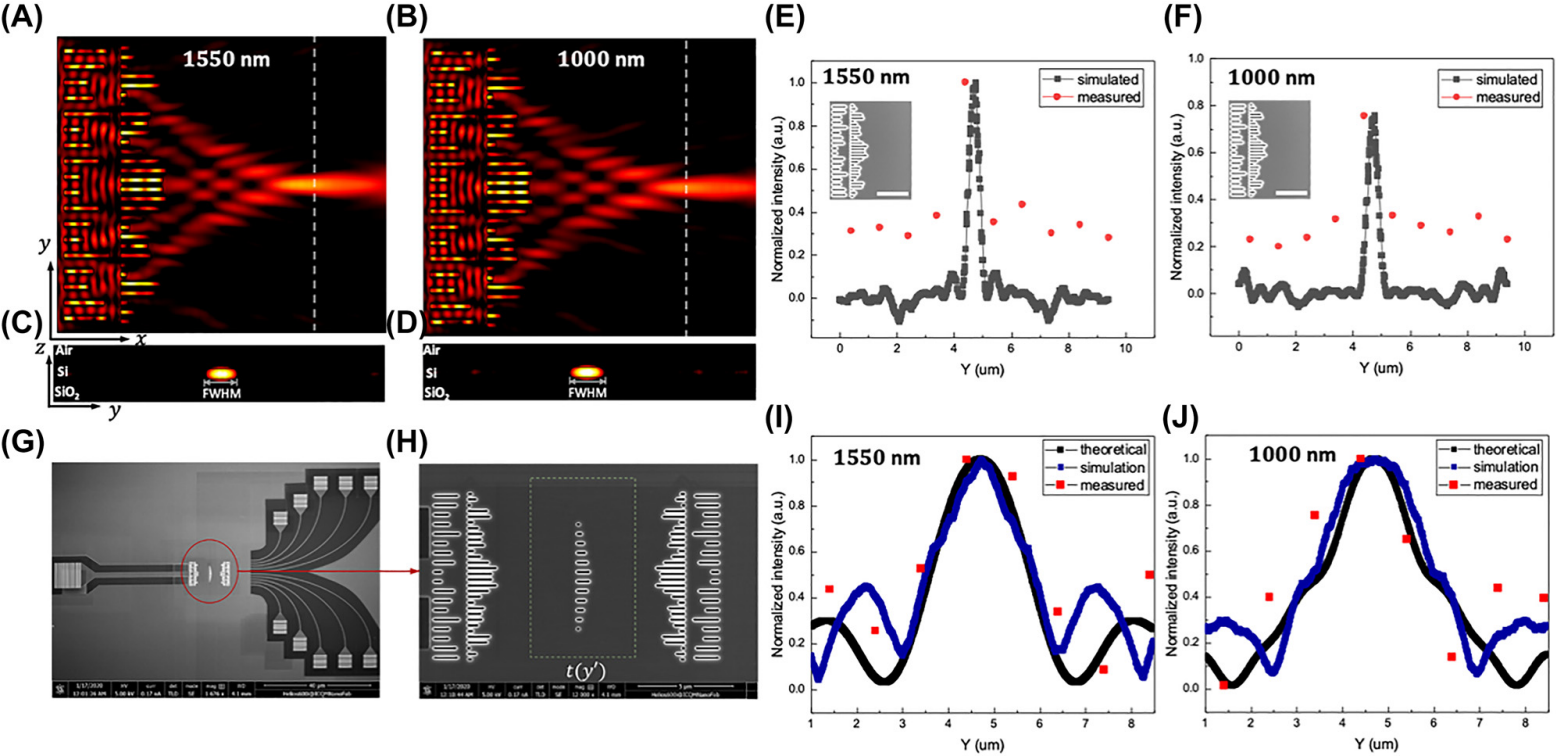
Broadband metasurface convolver [59]. (A) and (B) |*E*_y_|^2^, in plane (*x–y* plane) light distribution of the proposed dispersionless metalens at wavelength of 1550 and 1000 nm, respectively. The white dotted line shows the focal plane. (C) and (D) Cross-section (*y–z* plane) view of light distribution at the focal plane with wavelength of 1550 and 1000 nm, respectively. (E) and (F) Measured light intensity distribution on focal plane along the *y*-direction compared with simulation results at the dual wavelengths, respectively. Inset: the scanning electron microscope (SEM) image of the characteristic structure of the dispersionless metalens. The scale bar here is 3 μm. (G) The overall view of SEM image of the proposed convolver. (H) The characteristic structure of the nanophotonic convolver. The area enclosed by the middle dashed box is the given structure with its transmission function *t*(*y*′). (I) and (J) Measured spatial convolution results along the *y*-direction compared with simulation and theoretical results at the two wavelengths, respectively. The figures are reproduced with permission. Ref. [59] © 2020, The Author(s).

**Figure 5:**
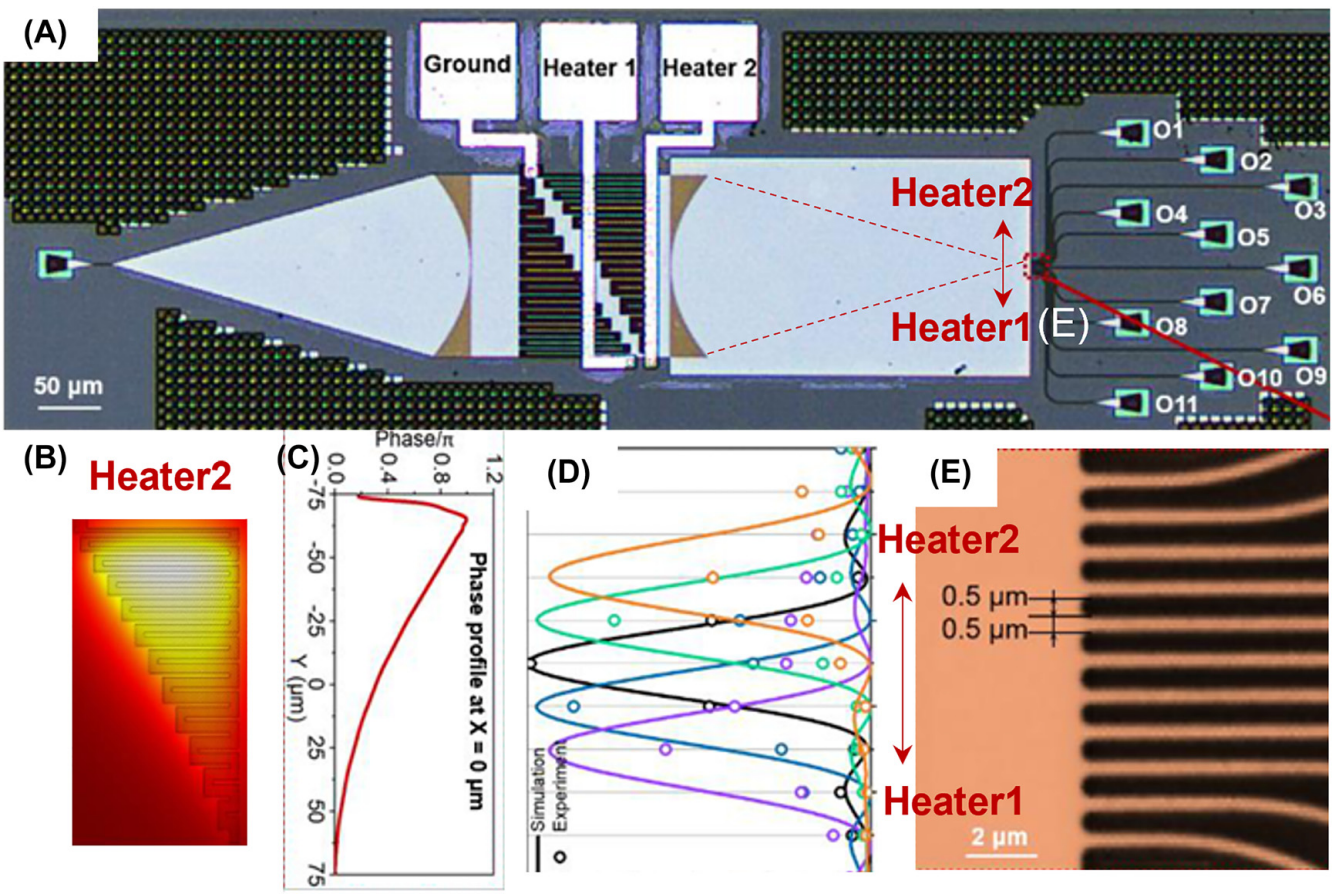
1 × *N* switch via on-chip tunable metasurface [85]. (A) The fabricated 1 × 11 switch by Advanced Micro Foundry (AMF). (B) Simulated temperature distribution by powering heater 2, and correspondent (B) wavefront of the transmitted wave incident on the second metalens. (C) The simulated and measured optical intensity profile of each output port on the focal plane. (D) Zoom-in optical image of the output ports on the focal plane.

The dispersionless metalens is realized by two air slot arrays with different periods etched in SOI substrate. The center of focal points for the two wavelengths are displaced less than 100 nm, far less than the nominal focal length of 8 μm, demonstrating the two focal planes can be considered the same to enable this system to perform parallel processing for dual wavelengths simultaneously (Figure 4A–D). The focusing efficiency is as high as 79 and 85% at wavelengths of 1000 and 1550 nm respectively (Figure 4E and F). Two sets of identical dispersionless metalens placed symmetrically about the focal plane form the 4*f* optical processing system. As shown in Figure 5G and H, a given structure with a certain transmission function is placed on the focal plane of the 4*f* system. And finally, the convolution result of the transmission function presented by the structure and the spatial waveform of the input signal is obtained at the output ports with the measured deviation of less than 8% (Figure 4I and J). This on-chip nanophotonic convolver provides the basis for more complex integrated all-optical computing tasks.

## 5 Metasystem for machine learning

In the recent years electronics-based deep neural network (DNN) hardware accelerators have been extensively studied in both academia and industry. To date, tremendous efforts from different technical stacks, ranging from device to circuit to architecture, have been devoted to improving DNN hardware performance. At the device level, DNN hardware can be fabricated on standard complementary metal-oxide-semiconductor (CMOS) [60–65] and emerging devices such as memristors [66–68] etc. At the circuit level, both the digital [69–71] and analog circuit design [72–74] can be adopted for implementation strategy. At the architecture level, the classical von Neumann architecture [61, 75] and emerging non-von Neumann architecture [74–78] can serve as the underlying architectural paradigm. Fulfilling DNN in the optical domain has received increasing attention recently. Without considering the phase corrections, ideal optical computing systems inherently offer massive parallelism and power efficiency. Here we compare the metasystem’s performance to the other integrated photonic architectures (Section 5.1) and free-space Fourier-optic image classifiers (Section 5.2).

### 5.1 Matrix operation in integrated metasurface system

Single-mode waveguides offers the best dispersion control and modal stability. It serves as the fundamental building blocks for broadband directional couplers (for bar, cross and power splitters) and microring resonators (wavelength multiplication). Circuit architectures for signal routing in photonics using cascaded Mach–Zehnder interferometers (MZI) to perform singular value decomposition (SVD) or Fourier transform (FT) is achieved with butterfly circuit topology. Temporal-spectral multiplications implement multi-wavelength modulation and summations [65, 79, 80]. Different from those approaches, the integrated metasurface platform expands the complexity of information processing *via* diffraction and in-plane free-space propagation, inheriting the integrated family for ‘imaging devices’ (such as multi-mode interferometers and star couplers) [81].

Vector-by-matrix multiplication (VMM) is one of the fundamental operations in the accelerator hardware [82]. Table 1 compares the VMM power efficiency, throughput, and footprint of integrated photonic circuits based on MZI, microring resonators (MRRs), and integrated metasystem.

**Table 1:** Integrated photonic frameworks for VMM.

Method for	Multi-wavelength modulation	Singular value	Fourier transform	Diffraction
matrix operation	and summation [79, 80, 87]	decomposition [83]	based [84]	equation [76]
Device architecture	MRR weight banks [79, 87] and directional couplers [80]	MZI [83]	MZI with butterfly-style mesh topology	Cascaded metasurfaces
Fabrication offset correction	Electro-optic tuning	Electro-optic tuning	Electro-optic tuning	Included during the training
Weight matrix	16 × 16 [80]	4 × 4 [83]	16 × 16	450 × 2
Footprint (mm^2^)	16 [80]	0.75 [83]	0.4	0.135
Throughput (Tb/s)	11 [87]	1 [83]	1.536 [84]	5–10
Insertion loss	27 dB [80]	Not reported	Not reported	15 dB (average)
Operational power	17 fJ per MAC	1 pJ per FLOP	1.7 × 10^4^ fJ per TOPS [84]	10^−5^ fJ per FLOP

Calculations of throughput and power consumption in Table 1 are detailed in references [76, 83, 84]. As shown in the table, compared with the other methods for VMM, the integrated metasystem has denser weight elements per footprint, along with lower operational power requirement. Such advantage in accordance with the multimode nature of the diffraction-based method, allows more information capacity. However, lack of tunability limits the usage of the integrated metasystem platform.

### 5.2 Image classifier

The integrated metasystem can also be designed for FT-based neural network. Compared with FT achieved with butterfly circuit topology, the FT achieved by integrated metalens requires no operation power and has a smaller footprint. Compared with the 2D metasystem in free space, the metasurface on the integrated platform also offers better mechanical robustness, with the advantages of lower insertion loss and feasible fabrications for multi-layer structures. Currently, the main technical challenge is the layout design of a large number of input/output (I/O) ports on an integrated photonic platform with tolerable phase distortions from nanofabrication. Theoretically, a 2D metasurface with subwavelength unit-cells owns significant computing capabilities. However, experimental implementation of such a system for machine learning has never been reported in telecommunication wavelength or infrared, but feasible if the fabrication or alignment errors are considered in the training process. Commercially available components (digital micromirror device, DMD or diffractive optical elements) have a typical cell number of 10^4^ to 10^6^. Single layer component has been utilized for high-accuracy image classifications. The integrated photonic platform can eliminate out-of-plane light diffraction, and thus result in orders of magnitude lower insertion loss compared to free-space optical systems (Table 2).

**Table 2:** Comparison of neuron networks-based image classifiers.

Neuron network	Convolution neural network [99]		Convolution neural network [100]	Diffraction neural network (metasystems)
Programmed layer(s)	One amplitude-only layer of DMD		One phase-only layer of diffractive optic elements	Phase-only layers of metasurface
Reconfigurable	Yes		No	No
Postprocessing	Required		Required	Maximal only
Hyperspectral	No		Possible	Yes
Kernel size	16 × 208 × 208		16 × 32 × 32	450 × 2
Dataset	MNIST	CIFAR	CIFAR-10	MNIST
Accuracy	98%(s)	63%(s)	51% (e)	96% (s)
Footprint	Bulk		Bulk	mm^2^

(s): Numerical simulation result. (e): experimental measurements.

## 6 Homogeneous multi-pixel switching schemes

Dynamic control of each individual pixel is critical for applications but challenging given high refractive index contrast is required for subwavelength light–matter interaction lengths. Conventional approach for integrated photonic device tunings, such as thermal-optic tuning, carrier injection or depletion, only introduces refractive index difference of around 10^−3^. Reprogrammable metasurface are achieved through tuning the refractive index of cladding liquid crystal [86] or bistable chalcogenide phase change materials [87]. Independent control of each metasurface unit-cell is usually required for full degree of freedom, which can be controlled by laser selective heating [80, 87] or arrays of liquid crystals [86]. Homogeneous change of refractive index, however, can also achieve a few special functions in metalens system with specialized material selection [88] or system design [85].

### 6.1 Electro-thermal tunability

**Figure 6:**
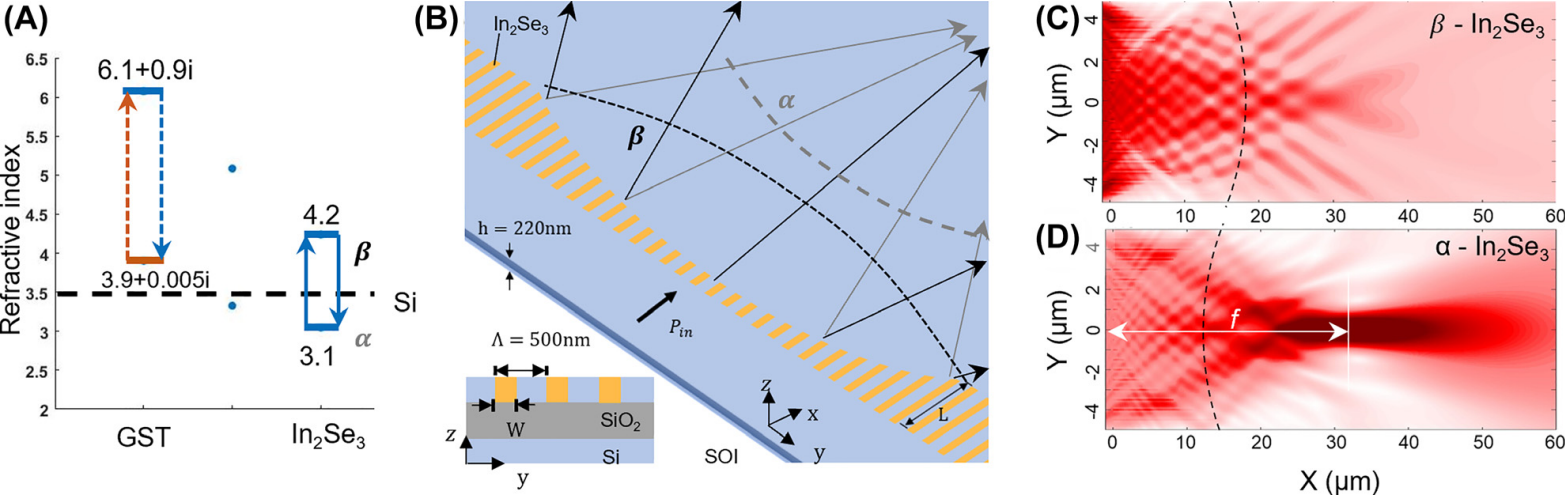
Phase change material embedded silicon photonic metalens [88]. (A) Refractive index comparison for the nonvolatile PCM materials at inter-convertible states/phase. (B) Perspective view of the PCM metalens on silicon photonic platform. For parallel light input, the wavefront of the transmitted wave is convex for α-state and concave for β-state. Arrows are the propagation directions, and the curves are the wavefronts of the transmitted light. Inset: cross-sectional view of the device. (C) Electrical intensity distribution for a metalens with β-In_2_Se_3_, and (D) 100% α-In_2_Se_3_. The dashed curves are the wavefronts.

Metasurface can also been used for high-performance optical switches with optimized heater design as shown in Figure 5A [85]. Focusing point of the metalens can be tuned using the thermo-optic effect. The effective index gradient in the silicon waveguide region caused by the designed temperature profile is shown in Figure 5B. The wavefront produces a phase difference along the *y* direction when the light wave propagates in the silicon waveguide under electrical power applied to the heater (Figure 5C). The focused beam can be steered towards upper and lower side by using heater 1 and heater 2, respectively. The simulated and measured optical intensity of each output port at the focal plane are plotted and compared in Figure 6D and E. The insertion loss is measured to be around 3–5 dB. The bandwidth exceeds 30 nm.

### 6.2 Focal length switching with embedded phase change materials

Optical phase-change materials (PCMs) offer an appealing material solution for active metasurface devices with their large index contrast and non-volatile switching characteristics [88]. Distinct from the other reported PCMs, the refractive index of silicon is near the middle of the ones for In_2_Se_3_ in two interconvertible states (Figure 6A), which means the two states introduce phase shift with opposite signs in an In_2_Se_3_ integrated silicon photonic metasurface [89]. For a designed metalens based on such gradient hybrid metasurface, homogeneous refractive index change of In_2_Se_3_ leads to dramatic focusing length tuning (Figure 6B). The hybrid integration of In_2_Se_3_ and silicon photonic does not need any additional lithography step but simple material deposition and standardized planarization. The amplitude of the electric field distribution exhibits clear defocusing (Figure 6C) and focusing behavior (Figure 6D) at 100% β and α-state, respectively. The wavefronts are extracted from the phase distributions at different β and α-states.

As the percentage of α-state reduces from 100 to 70%, the focusing length extracted from the curvature is comparable to the one estimated from the cross-sectional optical intensity profile at *y* = 0. Near the focal point (*x* = 3.2 μm, *y* = 0), the normalized light intensity reduces to 40% as the percentage of α-state reduces from 100 to 70%. The refractive index difference to In_2_Se_3_/silicon structure can be tuned from −0.4 to +0.7, resulting in focusing length variation from ≈30 to >50 μm, and from <−50 to ≈−25 μm. The reconfigurable transformative optical components have the potential for applications in miniaturized sensors and chip-scale nonlinear optics.

## 7 Perspectives

### 7.1 Applications in mode multiplexing and demultiplexing

**Figure 7:**
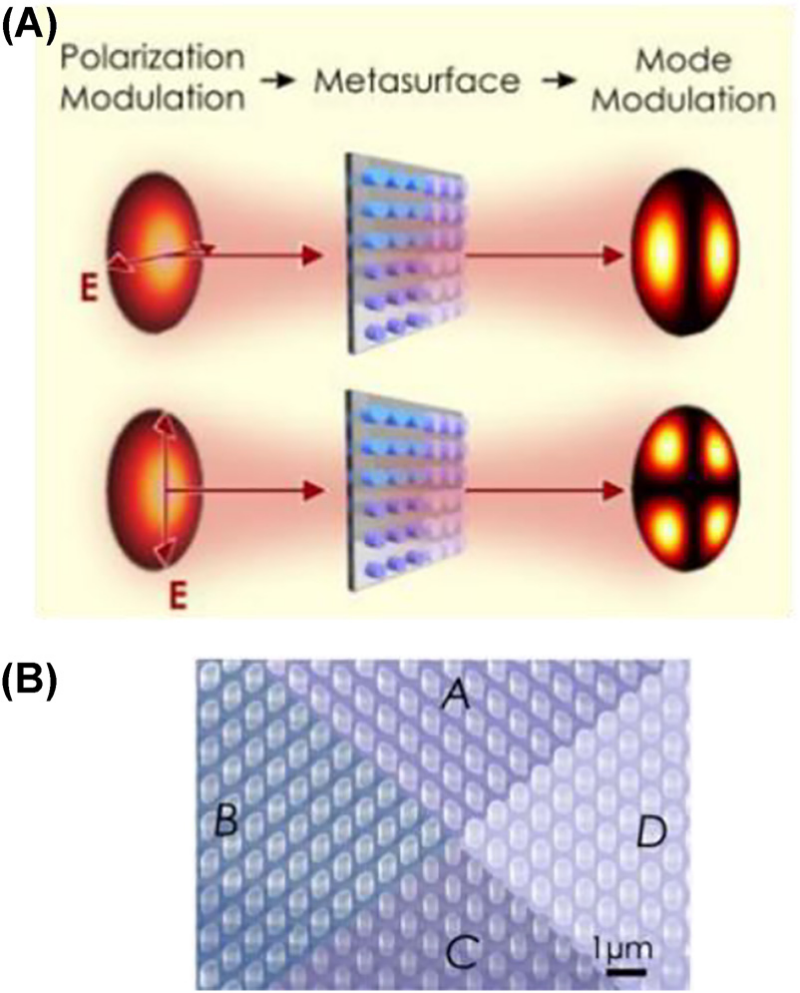
Metasurface generated OAM for the mode multiplexing in fiber and free-space optical communications. (A) Polarization modulation in metasurface. (B) SEM image of the metasurface [93, 94].

Within the past decades, advancements in the field of metasurface include (1) optimization and expanding the functionality of the metalens; (2) image processing and optical analog computing, including Fourier transform and differentiation; (3) chirality and polarization control; (4) reconfigurability [90]. In integrated optics, gradient metasurface along the waveguides control the mode-conversion and dispersion of guided waves [3, 91, 92]. Metasurfaces’ mode-conversion capabilities are useful in space division multiplexing for fiber and free-space communications [93]. Low insertion loss and broadband operation are of paramount importance. Arrays of dielectric metasurface devices have demonstrated spatial division multiplexing in fiber communications, which can simultaneously convert the two incomings orthogonally polarized LP01 signals into two different higher-order LP modes (LP11 and LP21), with operational bandwidth covering S-, C- and L-bands [93] (Figure 7A and B). A phase-only metasurface with a spiral phase pattern can convert the incoming Gaussian-like beam to a beam carrying orbital angular momentum (OAM). The metasurface-generated OAM beams have been utilized for optical free-space mode-division-multiplexing communications [94, 95]. Wavelength shift and parametric conversions are demonstrated with time-varying epsilon-near-zero metasurface [96–98]. The nonlinear response of the material introduces light-intensity dependent refractive index shift across and metasurface, resulting in ultrafast pulse shaping and wavefront engineering [97, 98].

Increasing data throughput demands, especially in short-distance communications within computer racks (meters) and between datacenters (kilometers), spatial information multiplication techniques beyond wavelength division multiplication (WDM) [101–103]. Multimode fibers and few-mode fibers offer a few spatial modes and orders of magnitude higher data capacity. On the transmitter end, vertical-cavity surface-emitting lasers generate two-dimensional spatial information with WDM [104]. On the receiver end, silicon photonic mode-division de-multiplexers have attracted attention with their CMOS compatibility and small footprint [2, 105, 106]. The development of low loss multi-mode optical components is distinguished from conventional silicon photonic device designs based on single mode waveguides. The profile of multi-mode waveguides, convertors, bends and cross bars need to be tailored in the way that multiple supported modes can operate with low loss [2]. Through controlling the on-chip wavefront, the dielectric metasurface systems offer compact and low loss designs for converting mode size [6] and mode orders [4]. With machine learning assisted designs, compression of spatial and spectral data information is possible through a cascaded metasurface system [107–109]. Beside the works mentioned above, there are some recent progresses towards tunable metamaterials, such as the vortex nanosieves to generate the optical beams carrying multiplexed OAM both in free-space and in a plasmonic near field [110], the metasurface that can be controlled by brainwaves [111], and nanostructures used for thermally stable solar thermophotovoltaic systems [112]. In [113, 114], the authors review the hyperbolic metamaterials and intelligent metasurfaces.

The integrated metasurface systems can serve as photonic front end for data dimension reduction. As the data rate in multimode fibers are in the scale of Tb/s, the data dimension reduction can reduce the amount of effective data flow and thus the bandwidth requirement of subsequent optoelectronic conversion. With rapid advance of silicon photonics, the typical radio-frequency (RF) – optical bandwidth for the silicon photonic modulators and detectors can reach tens of Gb/s. Reduction of data volume in all-optical formats can ease the device cost and power consumption in the optoelectronic conversion.

### 7.2 Individual meta-atom/cell programed by laser writing

**Figure 8:**
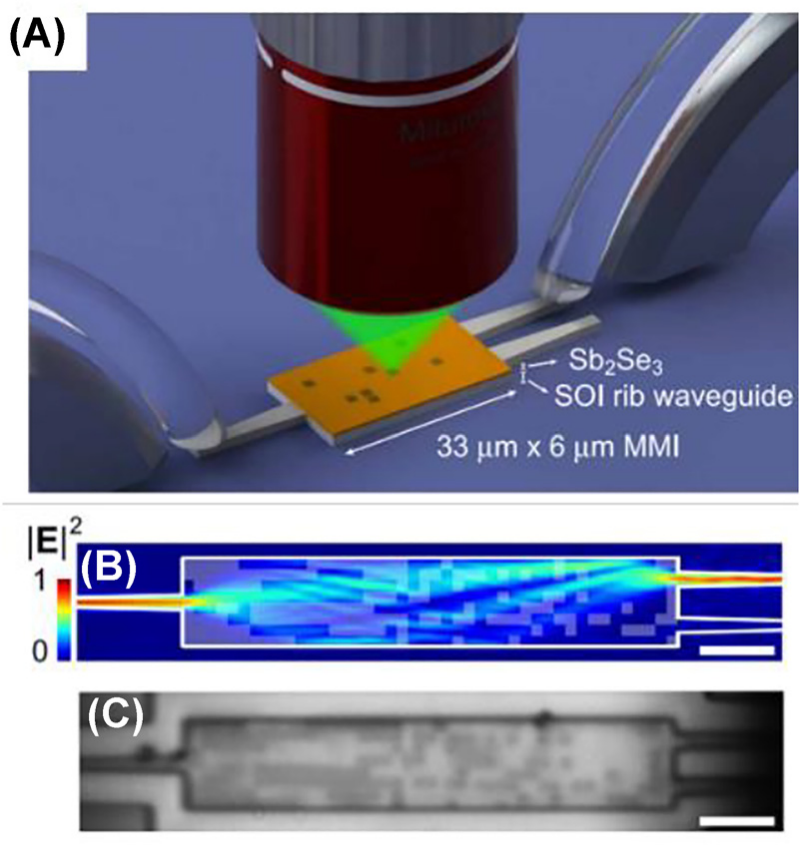
Perspective scheme for individual programming meta-atoms with vertically coupled laser. (A) Schematics of optical writing of patterns on a multimode interference router. (B) Numerically calculated electric field distribution in programmed device. The write area is the outline of the silicon device, and the white transparent patches represent the crystalline Sb_2_Se_3_ in amorphous background. (C) Optical microscope image of the laser programmed optical phase change material. Scale bars: 5 µm [81]. © 2021 The Authors.

The small spot size of lasers and high local intensity makes laser-writing a widely used tool. Similar to conventional laser writing lithography, lasers create patterns on chalcogenide materials through non-volatile phase transition. Those devices are low power and reconfigurable in the way that the laser exposed area will remain high refractive index contrast after laser removal. A simple baking resets the patterned film to be homogenous. Delaney et al. demonstrated the feasibility of a laser programmed PCM device (Figure 8) [80]. The localized programming can be applied to reconfigurable nonvolatile integrated photonic devices with minimal thermal cross talk. As an example, C. Choi et al. demonstrated a hologram technique through hybrid state Ge_2_Se_2_Te_5_ (GST) metasurface. Hybrid GST-Al metasurface structures are designed in the way that only at the specific middle states the holograph image is visible. The thermo-optical complexity in hybridized GST-Al metasurface allows the realization of secure cryptography [87].

## 8 Summary and perspectives

Given that on-chip dielectric metasurface offers a unique scheme to control degrees of freedom of the on-chip light field, as well as compatible with the standard CMOS fabrication process, it is expected to become one of the promising ways to realize on-chip integrated photonic computing chips with compact footprint, broadband, and low-loss on the basis of basic operations, like the convolution operation mentioned above, which is of great significance to promote the field of modern photonic technology in the future.

In summary, this paper reviews a variety of metasurface systems given its superior performance on spatial information processing with multiplication in spectral domains. As an arbitrary wavefront shaper, the low loss and broadband silicon photonic metasurface can serve as analog photonic accelerators and demultiplexer in MDM-DWDM optical communication. We review its role as Fourier optic mathematic operator for gradient metasurface. With machine learning accelerated reverse design, the metasurface system can perform advanced tasks such as image classification and hyperspectral imaging. Reconfigurability of such metasurface devices are still under development. With special designs and material selections, homogenous change of the refractive index can tune the focusing of the metalens. Individual unit can be reconfigured through local electrodes or laser exposure. Those active metasurface can play versatile and powerful roles in free space and fiber communications.
